# Berberine downregulates CDC6 and inhibits proliferation via targeting JAK-STAT3 signaling in keratinocytes

**DOI:** 10.1038/s41419-019-1510-8

**Published:** 2019-03-20

**Authors:** Shuna Sun, Xiaojie Zhang, Mengru Xu, Fang Zhang, Fei Tian, Jianfeng Cui, Yangyang Xia, Chenxi Liang, Shujie Zhou, Haifeng Wei, Hui Zhao, Guojing Wu, Bohan Xu, Xiaochen Liu, Guanqun Yang, Qinzhou Wang, Lei Zhang, Yaoqin Gong, Changshun Shao, Yongxin Zou

**Affiliations:** 1 Department of Dermatology, The Affiliated Hospital of Shandong University of Traditional Chinese Medicine, Shandong Provincial Hospital of Traditional Chinese Medicine, Jinan, 250011 Shandong China; 2 The Key Laboratory of Experimental Teratology, Ministry of Education and Department of Molecular Medicine and Genetics, Shandong University, School of Basic Medical Sciences, Jinan, 250012 Shandong China; 3 Department of Urology, Qilu Hospital, Shandong University, Jinan, 250012 Shandong China; 4 Department of Neurology, Qilu Hospital, Shandong University, Jinan, 250012 Shandong China; 5 Department of Immunology and Key Laboratory of Infection and Immunity of Shandong Province, Shandong University, School of Basic Medical Sciences, Jinan, 250012 Shandong China; 6 The First Affiliated Hospital of Soochow University and State Key Laboratory of Radiation Medicine and Protection, Institutes for Translational Medicine, Soochow University, Suzhou, 215123 Jiangsu China

## Abstract

Psoriasis is a chronic skin disease characterized by hyperproliferation and impaired differentiation of epidermal keratinocytes accompanied by increased inflammation, suggesting that molecules with antiproliferation and anti-inflammatory abilities may be effective for its treatment. One of the key steps in regulating cell proliferation is DNA replication initiation, which relies on prereplication complex (pre-RC) assembly on chromatin. CDC6 is an essential regulator of pre-RC assembly and DNA replication in eukaryotic cells, but its role in proliferation of keratinocytes and psoriasis is unknown. Here we examined CDC6 expression in psoriatic skin and evaluated its function in the proliferation of human keratinocytes. CDC6 expression is upregulated in epidermal cells in psoriatic lesions and it could be induced by IL-22/STAT3 signaling, a key signaling pathway involved in the pathogenesis of psoriasis, in keratinocytes. Depletion of CDC6 leads to decreased proliferation of keratinocytes. We also revealed that berberine (BBR) could inhibit CDK4/6-RB-CDC6 signaling in keratinocytes, leading to reduced proliferation of keratinocytes. The mechanism of antiproliferation effects of BBR is through the repression of JAK1, JAK2, and TYK2, which in turn inhibits activation of STAT3. Finally, we demonstrated that BBR could inhibit imiquimod-induced psoriasis-like skin lesions and upregulation of CDC6 and p-STAT3 in mice. Collectively, our findings indicate that BBR inhibits CDC6 expression and proliferation in human keratinocytes by interfering the JAK–STAT3 signaling pathway. Thus, BBR may serve as a potential therapeutic option for patients with psoriasis.

## Introduction

Psoriasis is a common chronic, recurring, and immune-mediated inflammatory skin disease, with a worldwide incidence of ~0.09–5.1% and seriously impairs the life quality of the patients^1–3^. A dysregulated crosstalk between epidermal keratinocytes and immune cells leads to inflammation, abnormal proliferation, and differentiation of keratinocytes, a hallmark of psoriasis^4–8^. The immune cells, which were mainly dendritic cells and T cells, infiltrating the skin lesions produce a large variety of cytokines such as interleukin (IL)-17, IL-22, IL-23, and IFN-γ that stimulate keratinocytes. On the other hand, activated keratinocytes can release numerous proinflammatory cytokines (e.g., IL-1, IL-18, TNF-α), chemokines, and antimicrobial peptides (AMPs) that can sustain psoriatic lesions^5–7^. Therefore, keratinocytes not only respond to psoriatic inflammation but also contribute to the recruitment and activation of immune cells. Thus, targeting keratinocyte proliferation and inflammation pathways can be used as effective therapies against psoriasis. However, the underlying mechanisms regulating these keratinocyte hyperproliferation remain largely elusive.

Although the molecular mechanisms involved in the pathogenesis of psoriasis are complex, growing evidence suggests that the activator of transcriptions 1 and 3 (STAT1 and STAT3), and nuclear factor-κB (NF-κB) is pivotal in the transcriptome network involved in the mechanism of psoriasis. STAT3 is an essential player to be responsible for the antibacterial/fungal type 3 (Th17) immune response and is considered to function as a central player in psoriasis pathogenesis^9,10^. STAT3 was reported to be active in psoriatic lesions, and suppression of STAT3 could inhibit proliferation and induce apoptosis of psoriatic keratinocytes^11^. In particular, expression of constitutively active STAT3 (STAT3C) in keratinocytes leads to the spontaneous development of psoriasis in transgenic mice^12,13^. Therefore, the targeting STAT3 pathway has been a promising target for the development of psoriasis therapies. Indeed, it was reported that STAT3 inhibitor not only inhibited the development of psoriasiform lesions in K5.Stat3C mice but also improved psoriatic lesions in psoriasis patients^14^.

CDC6 protein serves as one of the key regulators in DNA replication^15,16^. Interestingly, the recently published studies showed that CDC6 is also required for proper centrosome duplication^17,18^. Therefore, CDC6 is important for cell proliferation and is considered to be a specific biomarker of proliferating cells. CDC6 has been shown to be upregulated in tumors and associated with the progression and prognosis in various cancers^19,20^. However, the role of CDC6 in keratinocytes and psoriasis is unknown.

Currently, drug treatments such as retinoids, corticosteroids, and Vit D remain the main option for most psoriasis patients^4^. However, the efficacy of conventional drugs is limited because of adverse side effects and the development of pharmacoresistance^21^. Natural products are valuable sources in novel drug development. Berberine (BBR), a plant alkaloid, has been used for treating diarrhea and gastrointestinal disorders, particularly bacterial diarrhea, for thousands of years^22^. Clinical investigations on BBR revealed that it possesses various pharmacological effects, including antihyperglycemic, anticancer, and antidepressant^23^. However, the effect and molecular mechanisms of BBR in epidermal hyperplasia in psoriasis are unknown.

In the present study, we examined the expression of CDC6 in lesional skin of psoriasis and investigated its roles in keratinocytes. Our data indicated that CDC6, induced by STAT3 activation in keratinocytes, is upregulated in psoriatic epidermal skin and contributes to proliferation of keratinocytes. Moreover, BBR significantly inhibits CDK4/6-RB-CDC6 signaling, leading to cell cycle arrest and apoptosis in keratinocytes. We further found that BBR exerted its antiproliferation effects through inhibiting the JAK-STAT3 pathway. Importantly, BBR could inhibit the development of imiquimod-induced psoriasiform lesions and STAT3 activation in mice. These findings may have implications in designing therapeutic strategies for psoriasis.

## Material and methods

### Cell culture and manipulation

The immortalized human keratinocyte cell line HaCaT cells were cultured in Dulbecco’s Modified Eagle’s Medium (DMEM; Gibco, Carlsbad, CA, USA) supplemented with 10% fetal bovine serum (Gibco). The normal primary human epidermal keratinocytes (HEKn) were isolated from healthy skin biopsies as previously described^24^. HEKn were cultured in Epilife serum-free medium with human keratinocyte growth supplements added (Invitrogen, Carlsbad, CA, USA). All cells were maintained at 37 °C with 5% CO_2_. Plasmid transfections were performed using Lipofectamine 3000 (Thermo Fisher Scientific, Waltham, MA, USA), and siRNA transfections were performed using X-tremeGENE (Roche, Indianapolis, IN, USA) according to the manufacturer’s instructions. CDC6 and negative siRNA duplexes were purchased from Ribobio (Guangzhou, China). The sequence of siRNAs is listed in Supplementary Table 1. Generation of lentivirus and cells for overexpression of STAT3C was performed as described previously^25^. Berberine chlorid and MG132 were purchased from Sigma-Aldrich (Saint Louis, MO, USA). IL-6 and IL-22 were from R&D Systems (Minneapolis, MN, USA).

### IMQ-induced psoriasis-like skin inflammation in mice

Female BALB/c mice (8–11 weeks; 20–25 g) were obtained from Vital River Laboratory Animal Technology Co. Ltd (Beijing, China). All mice were bred and maintained under specific pathogen-free conditions. To construct a psoriasis-like skin inflammation mouse model, mice received a daily topical dose of 62.5 mg imiquimod (IMQ) containing cream (5%) (Aldara; 3M Pharmaceuticals) on the shaved back and 5 mg IMQ cream on the right ear for 5 consecutive days. Control mice were topically treated with vehicle Vaseline cream. For analyzing the effects of BBR, mice received Vaseline cream mixed with BBR (in DMSO solution) at a final concentration of 2 mM 5 h before topical application of IMQ creams on the shaved back and ear, once a day. The thickness of ears was measured using a digital caliper. After killing the mice, full thickness back and ear skin biopsies were collected. All experiments were performed in compliance with national regulations and approved by Animal Care and Use Committee, Shandong University, School of Basic Medical Sciences.

### Cell proliferation and migration assays

Cell proliferation was measured by CCK8 (Beyotime, Haimen, China) following the manufacturer’s protocols. Colony formation assays were performed as described previously^26^. Cell migration was evaluated by wound-healing and transwell migration assays as described previously^26^.

### Histopathological and immunohistochemistry analysis

The skin tissues were collected, fixed with formalin buffer, and embedded in paraffin. Hematoxylin and eosin (H&E) and immunohistochemical (IHC) staining was performed as previously reported^27^. The CDC6 staining intensity was visually scored using a four-value score for intensity (0 = negative, 1 = weak, 2 = moderate, and 3 = intense), and the percentage of scoring of staining was as follows: (0 = <10%, 1 = 10–29%, 2 = 30–59%, and 3 > 60% positive cells). An IHC expression score was obtained by multiplying the intensity and percentage values. The epidermal thickness measured in skin sections was quantified by a researcher blind to the experimental groups who took 10–15 random site measurements of the distance from the stratum corneum to the deepest part of the epidermis.

### EdU incorporation, cell cycle, and apoptosis assays

EdU (5-ethynyl-2′-deoxyuridine) incorporation and the cell cycle distribution analyzed by flow cytometry was carried out as described previously^26^. Cell apoptosis determined by Annexin V/PI double staining (Kaiji, Nanjing, China) was followed the manufacturer’s instructions. Briefly, cells were trypsinized, collected, and washed with PBS. Then cells were resuspended in 500 μl of binding buffer, and stained with annexin V-PE and PI for 30 min at room temperature. Cell fluorescence was measured on a Becton Dickinson FACScan instrument (BD Biosciences, San Jose, CA). TUNEL assays were performed as described previously^28^.

### RNA isolation and real-time PCR

Isolation of RNA and target mRNA expression analyzed by real-time quantitative PCR (qPCR) assays were performed as described previously^29^. The levels of GAPDH mRNA were used as endogenous control. All the experiments were performed in triplicate. Primer sequences used for qPCR are listed in Supplementary Table 2.

### Protein extraction and western blot

Total proteins were extracted with RIPA buffer and nuclear proteins were isolated with the Subcellular Protein Fractionation Kit (Beyotime, Haimen, China) following the manufacturer’s protocols. Mitochondria and cytosol proteins were extracted with the Mitochondria/Cytosol Fractionation Kit (Beyotime) following the manufacturer’s protocol. Western blot analysis was performed as described previously^26^. The primary antibodies are listed in Supplementary Table 3.

### Plasmids and luciferase assays

The constitutively active STAT3C lentiviral vector (plasmid #24983) was obtained from Addgene (Cambridge, MA, USA). Plasmid expressing HA-tagged full-length human CDC6 was a gift from L. Drury (Clare Hall Laboratories, Cancer Research UK, London, England)^30^. Luciferase reporter plasmid containing STAT3-responsive elements was obtained from Beyotime (Haimen, China). For luciferase assays, 0.2 µg STAT3 reporter construct was cotransfected with 0.02 µg of pRL-TK vector that provides constitutive expression of Renilla luciferase serving as an internal control. Six hours after transfection, cells were treated with or without BBR for another 24 h and the luciferase assays were performed using the Dual-Luciferase Reporter Assay system (Promega, Madison, WI, USA) and measuring with an LB960 Centro luminometer (Berthold Technologies, Germany). Transfections were performed in three independent experiments and assayed in quadruplicates. Data represent mean ± SD.

### ROS and JC-1 assay

Intracellular ROS levels were measured with the Reactive Oxygen Species Assay Kit (Beyotime) according to the manufacturer’s instructions. Briefly, cells were collected and resuspended in DMEM containing 10 µM DCFH-DA probe and incubated at 37 °C for 20 min. Then the cells were washed three times with PBS and measured with the FACScan instrument (BD Biosciences, San Jose, CA). The in situ detection of mitochondrial membrane potential (MMP, ΔΨm) was measured with the JC-1(5,5′,6,6′-Tetrachloro-1,1′,3,3′-tetraethyl-imidacarbocyanine iodide) assay kit (Beyotime) according to the manufacturer’s instructions. Briefly, cells were incubated with JC-1 containing buffer at 37 °C for 20 min, and then washed two times with iced JC-1 staining buffer. Afterward, cells were incubated in normal culture medium and visualized under a fluorescent microscope IX73 equipped with a DP80 digital camera (Olympus, Tokyo, Japan).

### Alkaline comet assay

Alkaline comet assays were performed using the Single Cell Gel Electrophoresis Assay Kit (Trevigen, Gaithersburg, MD, USA) according to the manufacturer’s protocol. Briefly, cells were collected and suspended in ice cold PBS cells and then combined with molten LMAgarose (at 37 °C) at a ratio of 1:10 (v/v). The mixture was immediately pipetted onto CometSlide™ and incubated at 4 °C in the dark for 10 min. Then the slide was incubated with 4 °C lysis solution for 30–60 min and electrophoresis was performed. The slides were stained with DAPI and viewed under a fluorescence microscope BX51 equipped with a DP71 microscope digital camera (Olympus).

### Statistical analysis

Data were analyzed using SPSS 13.0. Unless otherwise stated, differences between the mean values were analyzed for significance using the two-tailed unpaired *t*-test. *P* ≤ 0.05 was considered to be statistically significant.

### Study approval

Before the isolation of samples from patients, informed consent was obtained from each patient, with ethical approval received from Shandong Provincial Hospital of Traditional Chinese Medicine.

## Results

### CDC6 is upregulated in psoriatic skin and is required for keratinocyte proliferation

**Fig. 1:**
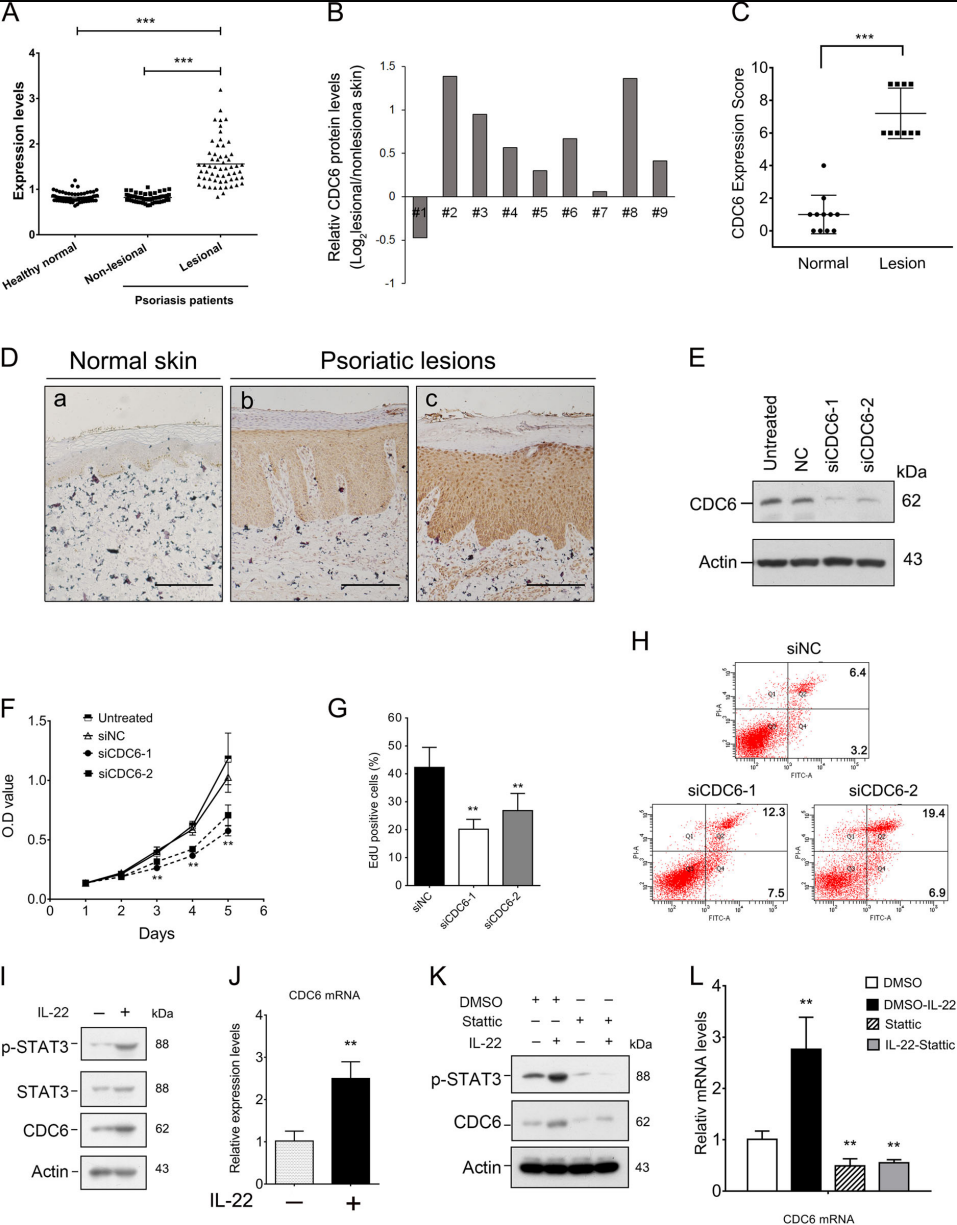
CDC6 is upregulated in psoriatic skin and is required for keratinocyte proliferation. **a** Expression analysis of CDC6 in clinical psoriasis samples using public GEO data (GEO ascension GDS4602). ***p < 0.001. **b** CDC6 protein levels in lesional skin of psoriasis relative to those in paired adjacent nonlesional skin were determined by optical density analysis of the results of western blot. **c** The statistical analysis of IHC staining for CDC6 in psoriatic lesional skin (10 patients) or healthy controls (11 subjects). Values represent the means ± SD. ***p < 0.001. **d** Representative IHC staining of CDC6 in the normal and psoriatic lesional plaque skin. Bar = 200 μm. Normal control skin with weak basal CDC6 expression (a), psoriatic lesional skin showing moderate (b), or intense full thickness CDC6 expression (c). **e** HaCaT cells were transfected with 50 nM negative control (NC) siRNA or CDC6 targeting siRNAs (siCDC6). Seventy-two hours later, the CDC6 protein levels were analyzed by western blot. **f**–**h** HaCaT cells were transfected with indicated siRNAs, and cell proliferation, DNA replication, and cell apoptosis were determined by CCK8 assays (F), EdU assays (G), and flow cytometry (H). **p < 0.01, compared to that of NC cells. **i, j** HaCaT cells were treated with 100 ng/ml IL-22 for 48 h and mRNA and protein levels of CDC6 were analyzed by western blot (I) and qRT-PCR (J). **p < 0.01, compared to that of untreated cells. **k, l** HaCaT cells were treated with or without 3 μM Stattic either alone or together with 100 ng/ml IL-22 for 48 h. CDC6 protein levels were determined by western blot (K), and mRNA levels of CDC6 were determined by qRT-PCR (L). **p < 0.01, compared to that of DMSO-treated cells

To explore the possible role of CDC6 in keratinocyte in psoriasis, we first examined expression of CDC6 in the affected skin of patients with psoriasis using the public GEO data set (GDS4602). The analysis showed that expression of CDC6 mRNA is similar in healthy normal and nonlesional skins of psoriasis patients, while it was significantly increased in lesional regions of psoriasis patients (Fig. 1a). Similar results were obtained using the GEO data set GDS3539 (Fig. S1a). Western blot was further employed to determine CDC6 protein levels, and the result showed that CDC6 protein levels were also increased in lesional skin of psoriasis (Fig. 1b, S1b).

To further evaluate whether CDC6 expression was upregulated in epidermis of psoriatic lesions, we performed immunohistochemistry on skin biopsies from patients with psoriasis and healthy individuals. CDC6 staining was very weak and predominantly distributed to the basal cell layer of the epidermis in healthy skin, and CDC6 was mainly expressed in the cytoplasm of epidermal keratinocytes in healthy samples (Fig. 1d, panel a). In contrast, CDC6 protein was higher in both cytoplasm and nuclear of keratinocytes and could be detected in all the epidermal layers in psoriatic lesional skin (Fig. 1c, d, panels b and c). Together, these results suggest that CDC6 is overexpressed in the epidermis of psoriatic lesions.

We then determined the role of CDC6 in DNA replication and proliferation of keratinocytes. The results showed that knockdown of CDC6 by siRNA significantly inhibited proliferation and DNA replication in HaCaT and the primary normal human epidermal keratinocytes (HEKn) cells (Fig. 1e–g, Fig. S1c–S1g). Moreover, RNAi of CDC6 induced apoptosis in HaCaT cells (Fig. 1h), indicating that CDC6 is required for proliferation of keratinocytes.

IL-22, released by T-helper-type 17 (Th17) cells and Th22 subsets, has been considered as a key cytokine that induces keratinocyte hyperproliferation in psoriasis^31–33^. We therefore evaluated the effects of IL-22 on CDC6 expression. The results showed that both protein and mRNA levels of CDC6 were upregulated in IL-22-treated keratinocytes (Fig. 1i, j, Fig. S1h,S1i). STAT3 is the principal mediator of IL-22 signaling psoriatic keratinocytes^31^. As expected, IL-22-treated cells showed a significant upregulation of the activated, Tyr705‐phosphorylated, form of STAT3 (Fig. 1i–k, Fig. S1h). Importantly, treated with STAT3 inhibitor Stattic antagonized the upregulation of CDC6 induced by IL-22 at both protein and mRNA levels (Fig. 1k, l), suggesting that the expression CDC6 can be induced in an IL-22/STAT3-mediated way in keratinocytes.

### Berberine downregulates the CDK4/6-RB-CDC6 pathway in keratinocytes

**Fig. 2:**
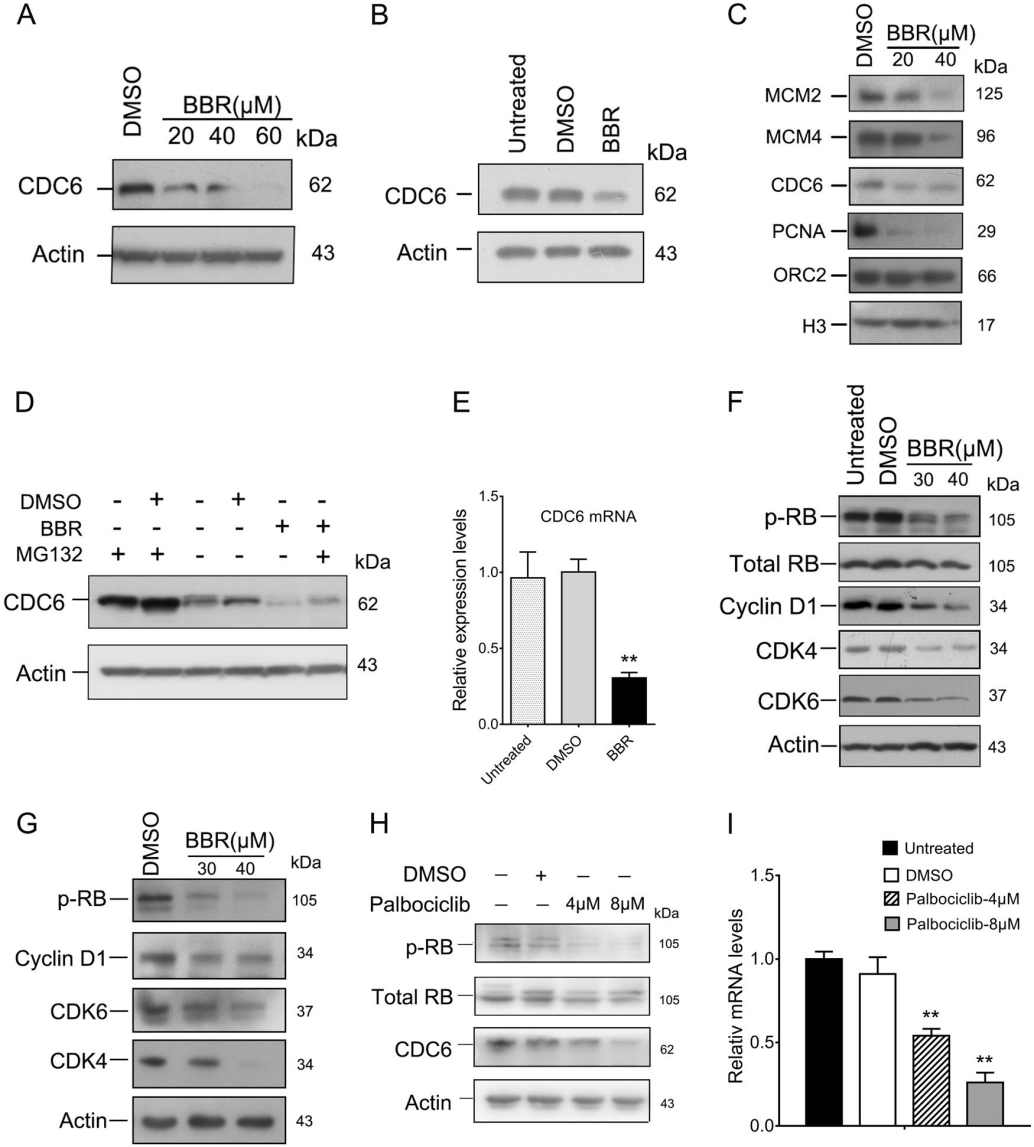
BBR downregulates the CDK4/6-RB-CDC6 pathway. **a** HaCaT cells were treated with or without BBR for 48 h and total proteins were analyzed by western Blot. **b** HEKn cells were treated with or without 40 μM BBR for 48 h and CDC6 expression was determined by western blot. **c** Chromatin bound proteins in HaCaT cells treated with or without BBR for 48 h were analyzed. **d** HaCaT cells were treated with 40 μM BBR for 48 h and then 8 μM MG132 for another 6 h. Cells were harvested and levels of CDC6 were determined by western blot. **e** HaCaT cells were treated with 40 μM BBR for 48 h and CDC6 mRNA levels were determined by qRT-PCR. **f, g** HaCaT (F) and HEKn (G) cells were treated with or without BBR for 48 h and indicated protein levels were analyzed by western blot. **h, i** HaCaT cells were treated with or without palbociclib for 48 h and then protein levels were measured by western blot (H) and mRNA levels were measured by qRT-PCR (I). **p < 0.01, compared to that of DMSO treated cells

As BBR has been reported to possess an anti-proliferative effect in cancer cells^23,34–37^, we next investigated the effects of BBR on expression of CDC6. Treatment of HaCaT cells with BBR resulted in a decrease in CDC6 protein levels in both HaCaT and HEKn cells (Fig. 2a, b). Binding of CDC6 to chromatin is essential for minichoromosome maintenance (MCM2-7) loading and therefore for DNA replication initiation^16^. As expected, chromatin binding MCM2 and MCM4 were significantly reduced in BBR-treated cells (Fig. 2c). Moreover, PCNA, a key factor necessary for DNA polymerase δ function, was also reduced (Fig. 2c). Together, these results suggest that BBR downregulates CDC6 expression in keratinocytes.

We next examined the mechanisms that BBR downregulates CDC6. As shown in Fig. 2d, the level of CDC6 was upregulated after MG132 treatment in BBR-treated cells, suggesting that MG132 inhibited proteasomal-mediated degradation of the existing CDC6 protein. However, the CDC6 protein level was still significantly decreased compared to that of DMSO- and MG132-treated cells, indicating that downregulation of CDC6 by BBR is not due to proteasome-mediated degradation. We then measured CDC6 mRNA levels. CDC6 mRNA was significantly downregulated in BBR-treated HaCaT and HEKn cells (Fig. 2e and Fig. S2), indicating that BBR may inhibit the transcription of CDC6 in keratinocytes. CDK4/Cyclin D kinase was reported to be important in CDC6 transcription^38^. We therefore determined levels of CDK4/6 and RB. As shown in Fig. 2f, g, BBR significantly downregulated proteins levels of CDK4/6, Cyclin D1, and phosphorylated RB in both HaCaT and HEKn cells. To substantiate that BBR downregulates CDC6 by inhibiting the CDK4-RB pathway, HaCaT cells were treated with CDK4/6 inhibitor palbociclib. As shown in Fig. 2h, i, palbociclib significantly reduced both protein and mRNA levels of CDC6. Together, these results suggest that BBR downregulates the CDK4/6-RB-CDC6 pathway in keratinocytes.

### Berberine inhibits proliferation and migration of HaCaT cells

**Fig. 3:**
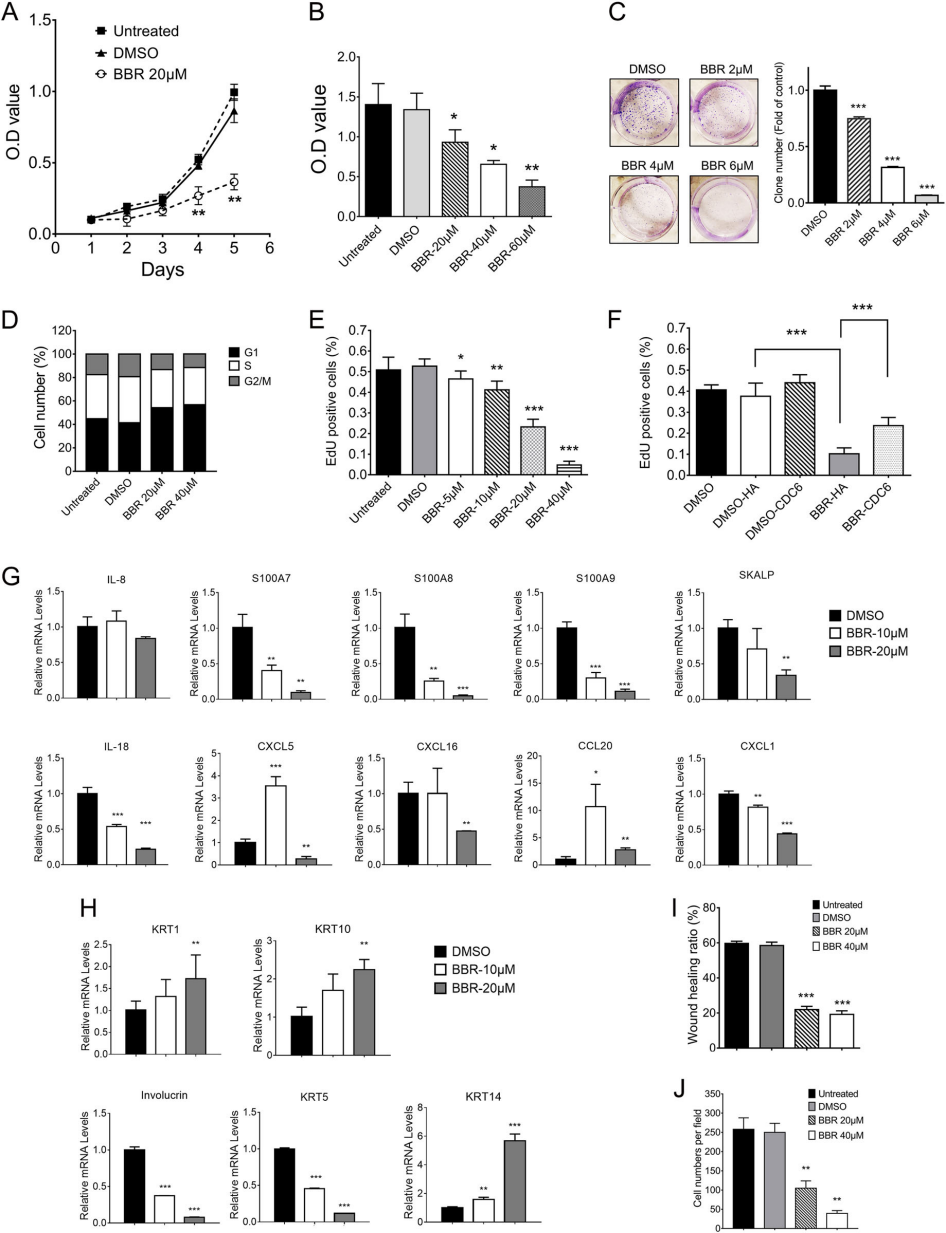
BBR inhibits proliferation and migration of HaCaT cells. **a** CCK8 assays of HaCaT cells treated with or without BBR. **b** CCK8 assays of HaCaT cells treated with BBR at different concentrations for 72 h. **c** Colony formation efficiency of HaCaT cells treated with or without BBR. **d** HaCaT cells were treated with or without BBR for 48 h and cell cycle distribution was analyzed by flow cytometry. **e** EdU assays of HaCaT cells treated with or without BBR for 24 h. **f** HaCaT cells were transfected with HA-tagged CDC6 expression plasmid or control plasmid (HA). Forty-eight hours later, cells were treated with or without 40 μM BBR for another 24 h, and EdU assays were performed. **g**, **h** HaCaT cells treated with or without 20 μM BBR for 48 h and indicated mRNA levels were measured by qRT-PCR. **i** HaCaT cells scraped with a pipette tip were treated with BBR for 24 h and the distances that the cells migrated were detected. **i** HaCaT cells were treated with BBR for 12 h, and transwell assays were performed. *p < 0.05, **p < 0.01, ***p < 0.001, compared to that of DMSO treated cells

We next evaluated the antiproliferative effects of BBR on keratinocytes. CCK8 assays showed that treatment with BBR resulted in a significantly slower growth rate compared to that of the untreated or DMSO-treated HaCaT cells (Fig. 3a). Moreover, the proliferative inhibitory effects of BBR were in a concentration-dependent manner (Fig. 3b). Similar results were observed in HEKn cells (Fig. S3a). Furthermore, clonogenicity analysis also showed that BBR-treated HaCaT and HEKn cells formed fewer and smaller colonies (Fig. 3c and Fig. S3b). The effect of BBR on cell cycle distributions was further analyzed. As shown in Fig. 3d, BBR treatment increased numbers of cells in G1 phase accompanied by a decrease in S fraction and G2/M phase in HaCaT cells. In contrast, no obvious change of cell cycle distribution was found in HEKn cells (Fig. S3c). EdU assays showed that EdU positive cells were significantly decreased in BBR-treated HaCaT and HEKn cells (Fig. 3e, Fig. S3d–S3f). Together, these results indicate that BBR inhibits proliferation of keratinocytes. We then determined the role of CDC6 in the inhibitive effects of BBR on HaCaT cells. Introduction of exogenous CDC6 restored the decreased CDC6 and partly rescued the decreased ability of EdU incorporation caused by BBR (Fig. 3f, S3g).

Next, we evaluated the effects of BBR on expression of antimicrobial peptides, proinflammatory cytokines, and chemokines associated with psoriasis. qPCR analysis showed that the mRNA levels of IL-18, S100A7/8/9, SKALP (Elafin), and CXCL1/16 were reduced with the increase in concentration of BBR in HaCaT cells, while no significant change in the levels of IL-8, CXCL5, and CCL20 was observed (Fig. 3g). We further investigated the effects of BBR on keratinocyte differentiation by detecting a differentiation keratinocyte marker. As shown in Fig. 3h, the mRNA levels of involucrin (IVL) and KRT5 were decreased, while KRT1, KRT10, and KRT14 were upregulated in BBR-treated HaCaT cells.

We then investigated the effects of BBR on migration. Wound-healing assays showed that BBR significantly suppressed wound closure in a concentration-dependent manner in both HaCaT and HEKn cells (Fig. 3i, S3h–S3j). In addition, the average number of migrating cells penetrating the transwell membrane was considerably lower in BBR-treated cells (Fig. 3j, S3k).

### Berberine induces apoptosis and oxidative DNA damage in HaCaT cells

**Fig. 4:**
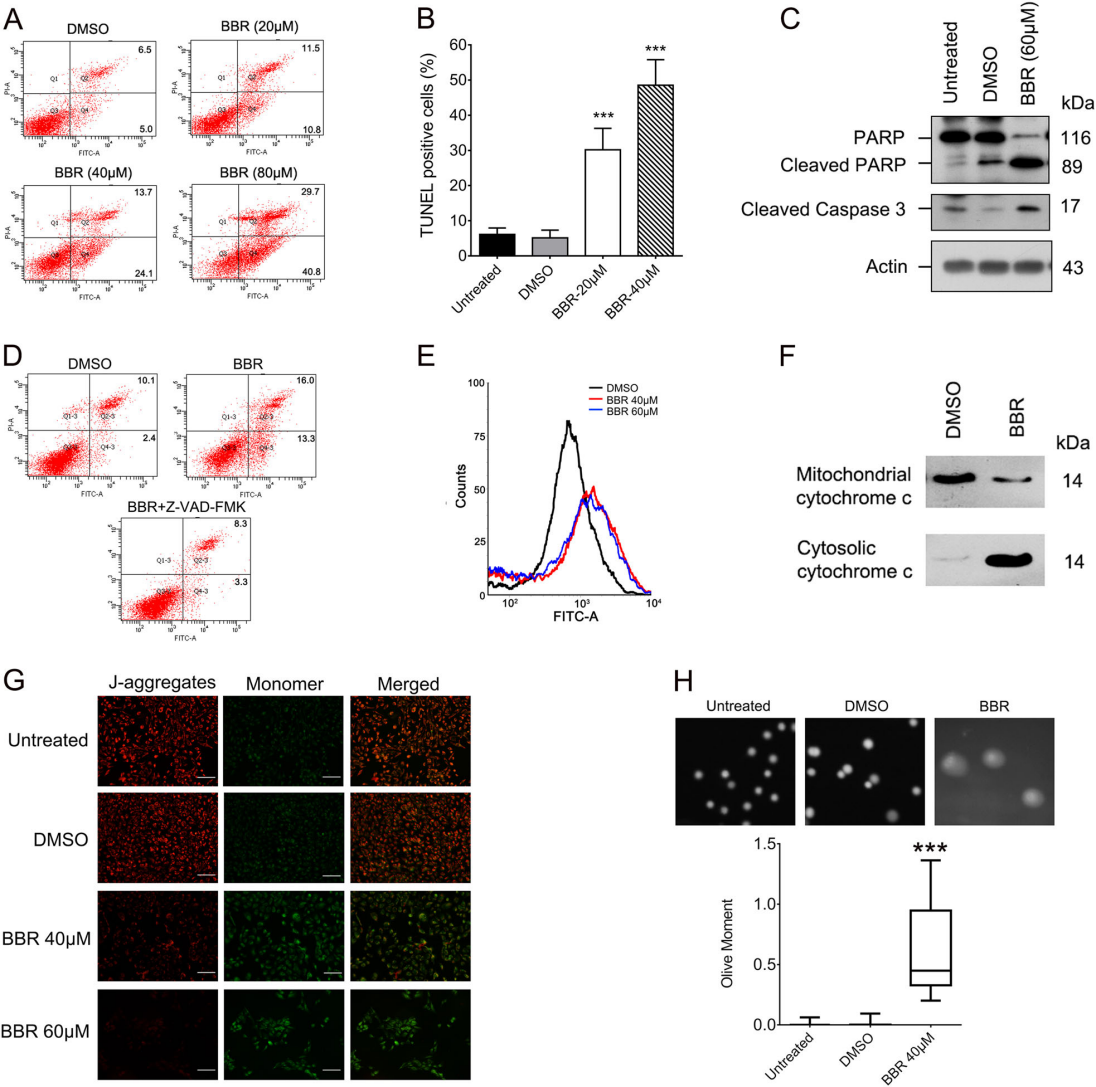
Berberine induces apoptosis and oxidative DNA damage in HaCaT cells. **a–c** HaCaT cells were treated with or without BBR for 72 h and cell apoptosis was assessed by flow cytometry (A), TUNEL assays (B), and western Blot (C). **d** HaCaT cells were pretreated with or without Z-VAD-FMK for 2 h followed by 40 μM BBR for 48 h and cell apoptosis was measured by flow cytometry. **e** HaCaT cells were treated with BBR for 24 h and ROS levels were determined by flow cytometry using DCFH-DA probe. **f**–**g** HaCaT cells were treated with or without BBR for 48 h, and mitochondria and cytosol cytochrome c levels were analyzed by western blot (F) and the mitochondrial membrane potential was measured by JC-1 assays (G). Scale bar, 100 μm. **h** Comet assays of HaCaT cells treated with or without 40 μM BBR for 48 h. ***p < 0.001, compared to that of DMSO-treated cells

We then investigated whether BBR causes apoptosis in keratinocytes. Flow cytometry assays showed that the numbers of apoptotic HaCaT and HEKn cells were significantly increased after 72-h treatment with BBR (Fig. 4a, S4a). Moreover, the number of TUNEL positive cells was also significantly increased in BBR-treated HaCaT cells (Fig. 4b, S4b). Furthermore, the protein levels of cleaved caspase-3 and PARP were significantly increased in BBR-treated HaCaT cells (Fig. 4c). To evaluate the significance of caspase activation in BBR-induced apoptosis, Z-VAD-FMK, a broad-spectrum caspase inhibitor, was added to the culture medium before BBR treatment. Pretreatment with Z-VAD-FMK efficiently blocked the apoptosis induced by BBR (Fig. 4d), indicating that BBR induces apoptosis through a caspase-dependent pathway.

A number of studies have reported that BBR induces DNA damage and cell apoptosis through production of reactive oxygen species (ROS)^39^. As shown in Fig. 4e, treatment of BBR also caused an increase in ROS levels in HaCaT cells. Mitochondria are the primary generators of ROS and intracellular ROS may induce disruption of the MMP and release of cytochrome c from mitochondria into the cytosol, which causes apoptotic cascades and eventually cell apoptosis^40^. As shown in Fig. 4f, g, treatment with BBR resulted in a release of cytochrome c into cytosol and a decrease in MMP, indicating that BBR induces the disruption of the mitochondrial. Comet assays were further conducted to measure DNA damage. As expected, BBR treatment significantly increased the mean olive tail moment value (Fig. 4h). Together, these results suggest that BBR increases the ROS level, disrupts mitochondrial, causes DNA damage, and induces apoptosis in keratinocytes.

### BBR downregulates STAT3 activity in HaCaT cells

**Fig. 5:**
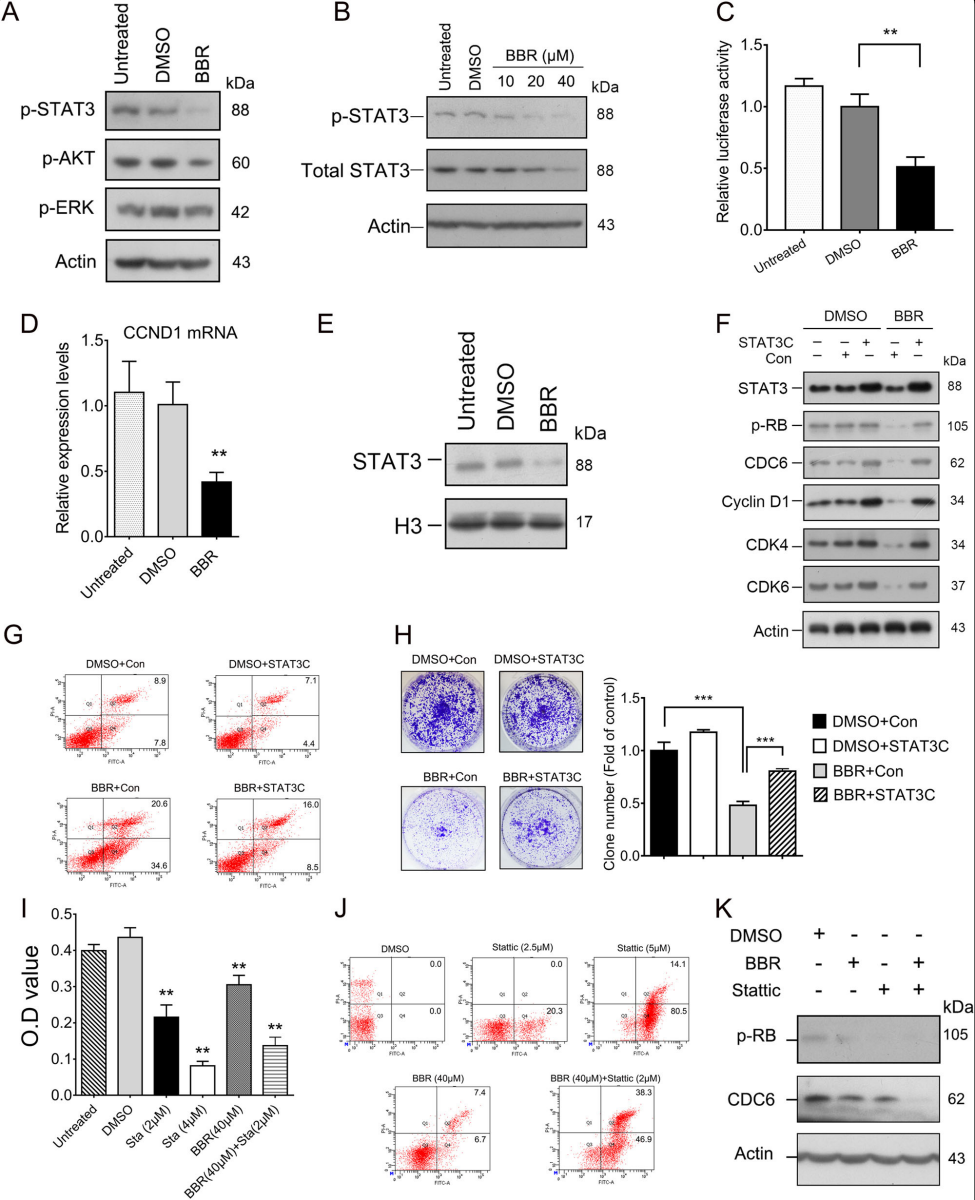
BBR downregulates STAT3 activity in HaCaT cells. **a** HaCaT cells were treated with or without 40 μM BBR for 48 h and levels of indicated proteins were determined by western blot. **b** HaCaT cells were treated with different concentrations of BBR and indicated protein levels were analyzed by western blot. **c** HaCaT cells were transfected with STAT3 responsive luciferase reporter. After 24 h, cells were treated with or without 40 μM BBR for another 24 h and luciferase assays were performed. **d, e** HaCaT cells were treated with or without 40 μM BBR for 48 h; CCND1 mRNA levels were determined by qRT-PCR (D) and nuclear STAT3 levels were determined by western blot (E). **f–h** HaCaT cells expressing STAT3C or control GFP lentivirus (Con) were treated with or without 40 μM BBR for 72 h, and protein levels were determined by western blot (F), and cell apoptosis was measured by flow cytometry (G). Cell proliferation was determined by colony formation assays (H). **i, k** HaCaT cells were treated with or without Stattic either alone or together with BBR for 48 h. Cell viability was detected by CCK8 assays (I); cell apoptosis was detected by flow cytometry (J) and protein levels were determined by western blot (K). **p < 0.01, ***p < 0.001, compared to that of DMSO-treated cells

To further investigate the mechanism that BBR inhibits proliferation of keratinocytes, activation of AKT, ERK, and STAT3, which is known to be important cell survival pathways, was examined. The results showed that BBR treatment resulted in a significantly decreased level of p-705-STAT3 and moderately decreased p-308-AKT (Fig. 5a).

The STAT3-mediated pathway plays an important role in psoriasis^12^; we then determined the effect of BBR on STAT3. Western blot analysis showed that the inhibitory effects of BBR on the phosphorylation levels of STAT3 were in a concentration-dependent manner in both HaCaT and HEKn cells (Fig. 5b, S5). Luciferase assays showed that the STAT3 transcription activity was significantly decreased by BBR (Fig. 5c). Moreover, mRNA levels of CCND1, the target of STAT3, were also decreased by BBR (Fig. 5d). Activated STAT3 is translocated from cytoplasm to the nucleus and induces the transcription of downstream genes; we therefore detected the levels of nuclear STAT3 after BBR treatment. As expected, the nuclear STAT3 was decreased in BBR-treated cells (Fig. 5e). Together, these results suggest that BBR inhibits the transcription activity of STAT3 in HaCaT cells.

We next sought to determine whether STAT3 was responsible for BBR-mediated proliferation inhibition and apoptosis. Expression of constitutively active STAT3(3C) constructs in HaCaT cells restored the BBR-induced decrease in CDK4/6, Cyclin D1, p-RB, and CDC6 levels (Fig. 5f), and partially restored the apoptosis and proliferation inhibition induced by BBR (Fig. 5g, h). In addition, similar to that of BBR, STAT3 inhibitor Stattic treatment also resulted in proliferation inhibition, apoptosis, and downregulation of p-RB and CDC6 in HaCaT cells (Fig. 5i–k). These results indicated that STAT3 is a key target through which BBR exerts its proliferation inhibition effects on keratinocytes. It should be noted that STAT3C showed only partial rescue effects (Fig. 5g, h), and Stattic enhanced the effects of BBR on downregulation of p-RB, CDC6, proliferation inhibition, and apoptosis (Fig. 5i–k), suggesting that, in addition to STAT3, BBR also acts on multiple other targets in keratinocytes.

### BBR perturbs JAK-STAT3 signaling in keratinocytes

**Fig. 6:**
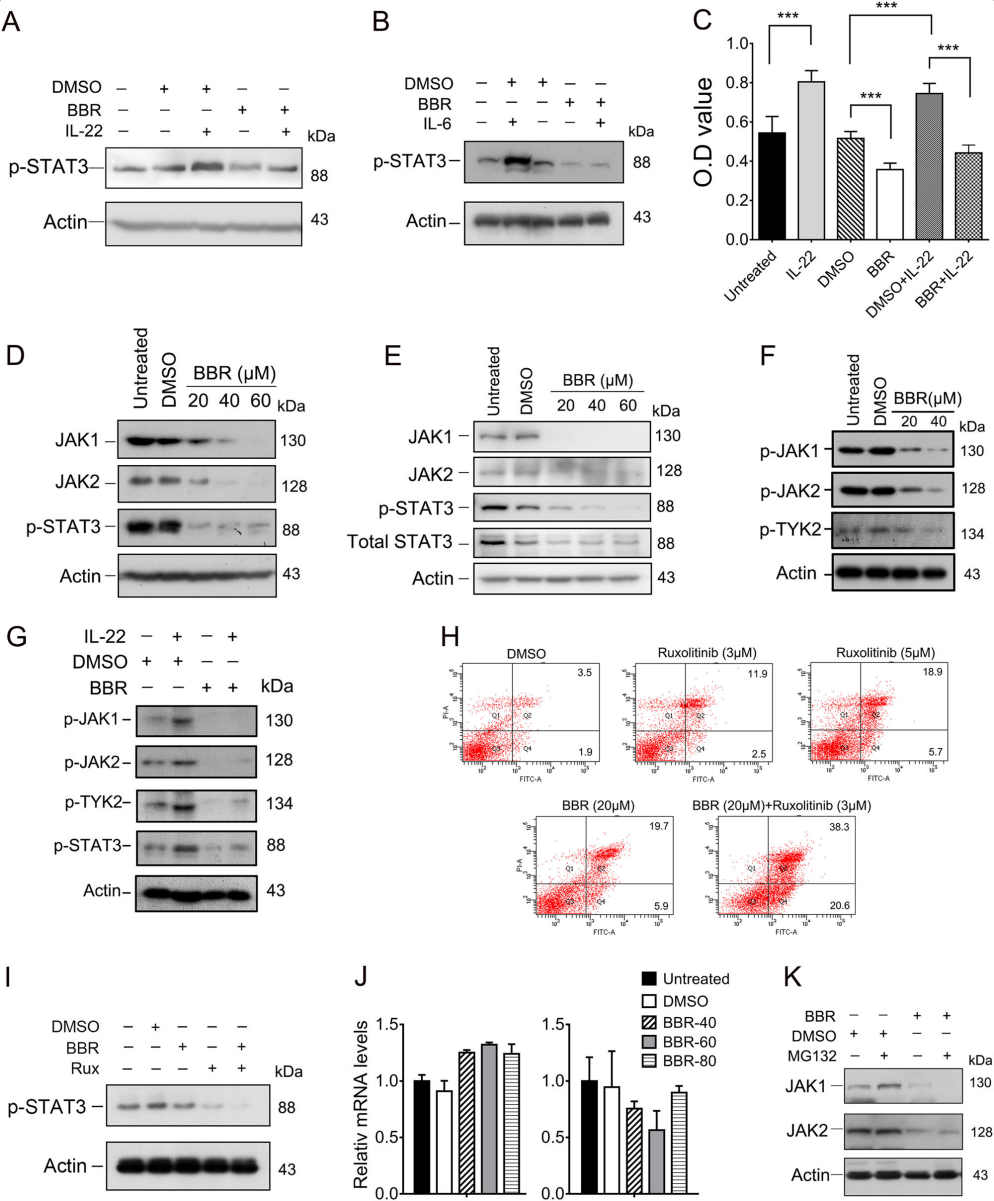
BBR inhibits STAT3 activation by downregulating JAK in HaCaT cells. **a, b** HaCaT cells were treated with or without 40 μM BBR for 24 h followed by treatment with 100 ng/ml IL-22 (A) or IL-6 (B) for another 24 h and indicated protein levels were determined by western blot. **c** HaCaT cells were treated with or without 20 μM BBR for 24 h followed by treatment with 100 ng/ml IL-22 for another 24 h, and cell proliferation was determined by CCK8 assays. ***p < 0.001. **d, e** HaCaT (D) and HEKn (E) cells were treated with or without BBR for 48 h and indicated protein levels were analyzed by western blot. **f** HaCaT cells were treated with or without BBR for 48 h and indicated protein levels were analyzed by western blot. **g** HaCaT cells were treated with or without 20 μM BBR for 24 h followed by treatment with 100 ng/ml IL-22 for another 24 h and indicated protein levels were determined by western blot. **h** HaCaT cells were treated with or without ruxolitinib either alone or together with BBR for 48 h. Cell apoptosis was detected by flow cytometry. **i** HaCaT cells were treated with or without 3 μM ruxolitinib either alone or together with 10 μM BBR for 24 h. Protein levels were determined by western blot. **j** HaCaT cells were treated with 40 μM BBR for 48 h and indicated mRNA levels were determined by qRT-PCR. **k** HaCaT cells were treated with 40 μM BBR for 48 h and then MG132 for another 6 h. Cells were harvested and indicated protein levels were determined by western blot

As STAT3 can be activated by cytokine stimulation, we then evaluated whether BBR could antagonize STAT3 activation induced by IL-22 and IL-6 in keratinocytes. BBR administration reverted the upregulation of STAT3 phosphorylation induced by IL-22 and IL-6 (Fig. 6a, b), suggesting that BBR inhibits cytokine-mediated activation of STAT3. We then determined whether BBR could perturb IL-22-induced keratinocytes hyperproliferation. As expected, IL-22 stimulation promoted proliferation of HaCaT cells. However, this effect was markedly reversed by BBR (Fig. 6c).

IL-6 and IL-22 bind to a receptor and the signal transduction occurs via Janus kinases (JAKs), which in turn promotes STAT3 phosphorylation and activation. To elucidate the mechanisms underlying the inhibition of STAT3 by BBR, we examined the expression of JAK1, JAK2, and TYK2. BBR significantly decreased the total protein levels of JAK1 and JAK2 in HaCaT and HEKn cells (Fig. 6d, e and Fig. S6a). We further investigated whether the JAK activity is affected by BBR in keratinocytes. As shown in Fig. 6f, g and Fig. S6b, c, BBR treatment decreased both basal- and IL-22-induced JAK1/2 and TYK2 tyrosine phosphorylation level. Moreover, BBR significantly enhanced the selective JAK1/2 inhibitor, Ruxolitinib, induced apoptosis, and decrease of STAT3 phosphorylation (Fig. 6h, i). Together, these results indicated that BBR perturbs JAK-STAT3 signaling in keratinocytes.

We next investigated the mechanisms that BBR downregulates JAK1/2 expression. BBR treatment did not downregulate mRNA levels of JAK1/2 (Fig. 6j). Moreover, treatment with MG132 could not block the decreased expression of JAK1/2 proteins induced by BBR (Fig. 6k), suggesting that BBR did not affect the proteasome-mediated degradation of JAK1/2.

### BBR protects against IMQ-induced psoriasis-like skin lesion

**Fig. 7:**
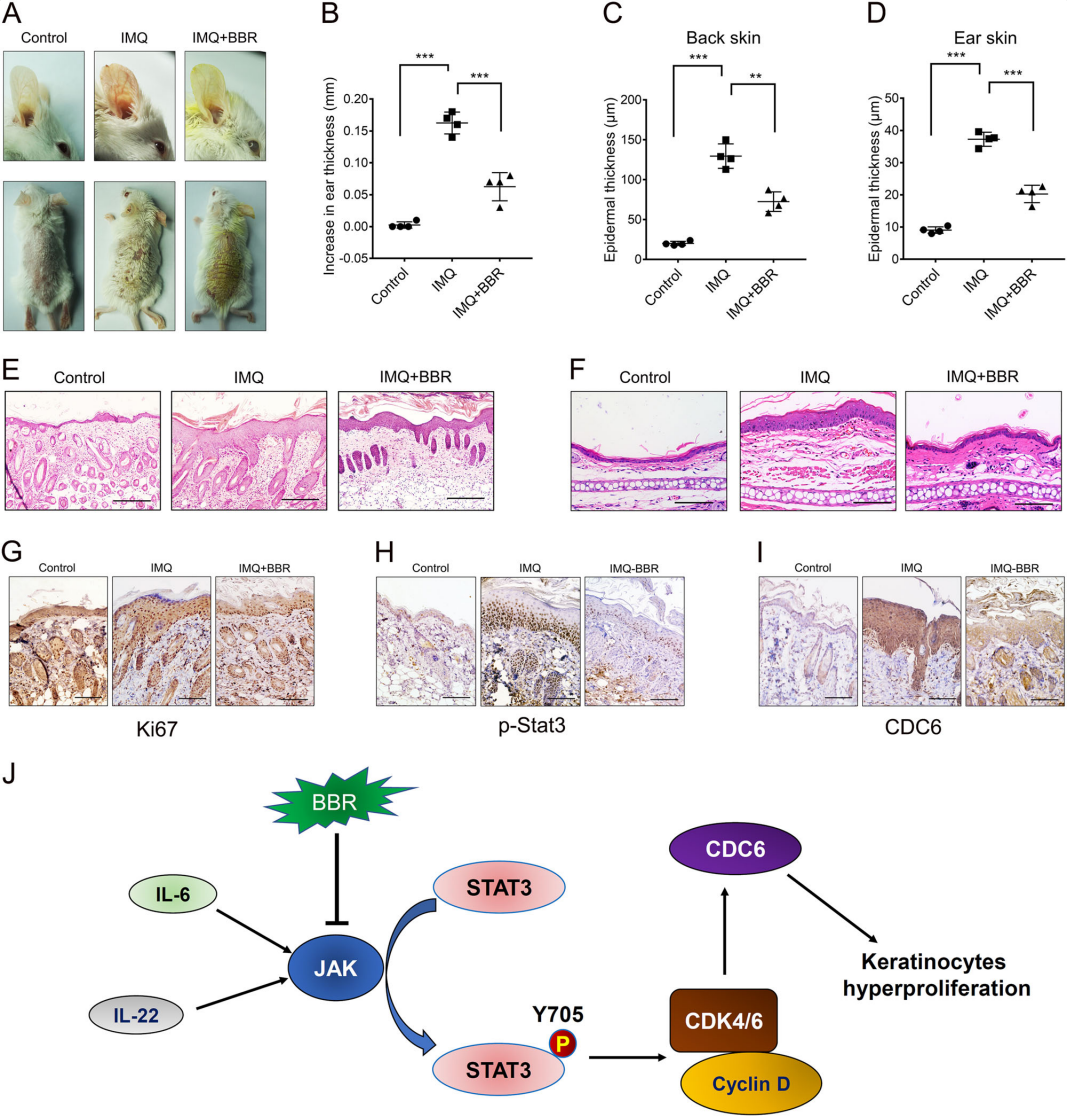
BBR alleviates IMQ-induced psoriasis-like skin lesion in the mouse model. **a** Representative images of mice treated with vehicle (control), IMQ or IMQ together with BBR for 5 days. For each group, *n* = 4. **b** Analysis of the increase in ear thickness in different groups after treatment with vehicle, IMQ or IMQ together with BBR for 5 days. Each point represents one mouse. **c, d** Analysis of epidermal thickness of back (C) and ear (D) skin in different groups. **e, f** Representative histological sections of the back (E, bar = 200 μm) and ear (F, bar = 100 μm) skins of mice. **g–i** IHC staining of Ki67 (G), p-STAT3 (H), and CDC6 (I) in back skin of different groups, bar = 100 μm. **j** Schematic model of the mechanisms by which BBR downregulates CDC6 and inhibits proliferation in human keratinocytes. P, phosphate

We then evaluated the therapeutic effects of BBR ‘in vivo’ in the IMQ-induced murine model. IMQ application for 5 days significantly increased the ear and epidermal thickness compared with that of the control group (Fig. 7a–f). However, the ear and epidermal thickness was significantly reduced in the IMQ and BBR co-treatment group when compared with the IMQ-treated group (Fig. 7a–f). Moreover, the proliferation marker Ki67 in epidermis was also markedly decreased by BBR treatment (Fig. 7g). Together, these data indicate that BBR effectively suppresses IMQ-induced psoriasis-like inflammation and epidermal hyperproliferation in mice. We further investigated the effects of BBR on expression of p-STAT3 and CDC6. IMQ treatment significantly upregulated the expression of p-STAT3 and CDC6 in epidermis (Fig. 7h, i). However, this effect was reduced when coadministered with BBR (Fig. 7h, i).

## Discussion

Epidermal hyperproliferation is a hallmark of psoriasis, and abnormal proliferation and differentiation of keratinocytes, the most predominant cell type of the epidermis, are the key events involved in psoriasis development^8^. Therefore, inhibiting keratinocyte proliferation and induced apoptosis can be used as effective therapeutic agents against psoriasis. In the present study, we demonstrated that CDC6 expression in keratinocytes could be enhanced by IL-22/STAT3 signaling, which is involved in initiation and maintenance of the pathogenesis of psoriasis^31^. Silencing of CDC6 resulted in reduced proliferation and DNA replication, and induced cell apoptosis of keratinocytes. More importantly, we showed that both mRNA and protein levels of CDC6 were upregulated in epidermal keratinocytes from lesional skin of psoriasis. Therefore, CDC6 may be responsible for the aberrant proliferation of keratinocytes in psoriasis and could be targeted for the treatment of psoriasis.

Until now, very little is known about the roles of BBR in keratinocytes. It has been recently reported that BBR inhibits proliferation and migration, induced apoptosis in the epidermoid carcinoma cell A431^41^. Here, we observed that BBR significantly decreased CDC6 expression and inhibited pre-RC assembly through inhibiting the CDK4/6-RB pathway in keratinocytes (Fig. 7j). Furthermore, BBR significantly inhibits proliferation and migration of keratinocytes. We also demonstrated that BBR treatment results in ROS generation, loss of MMP, and release of cytochrome c from mitochondria and ultimately leads to cell apoptosis. Moreover, BBR inhibits IMQ-induced psoriasis-like skin lesion in mice and significantly downregulates the phosphorylation level of STAT3 in keratinocytes and IMQ-induced murine epidermis. As BBR has been shown to be relatively safe to normal cells in the majority of laboratory and clinical situations^42,43^, our results here suggest that BBR is a potential agent for combating psoriasis. It is worth mentioning that previous studies showed that BBR inhibited differentiation of Th17 cells and decreased phosphorylation of STAT3 in differentiating Th17 cells^44,45^, which are prominently involved in the pathogenesis of psoriasis^46^. Therefore, BBR could inhibit STAT3 in both immunocytes and keratinocytes that are implicated in psoriasis development.

Our results demonstrated that STAT3 is a key target of BBR, through which BBR exerts its effects on keratinocyte proliferation inhibition (Fig. 7j). IL-22 is a key pathogenic cytokine in psoriasis and the main mediator of IL-22 signaling is STAT3^31–33^. Here, we demonstrated that BBR effectively reverted STAT3 phosphorylation and keratinocyte hyperproliferation induced by IL-22, suggesting that BBR could block the IL-22-STAT3 pathway in keratinocytes. BBR was shown to inhibit STAT3 activation in cancer cells and CD4^+^ T cells^44,47–51^. However, the underlying mechanisms are still poorly understood. JAKs belong to the group of cytoplasmic tyrosine kinases that are essential upstream STAT activators^9^. Therefore, inhibition of JAKs could abrogate STAT activation induced by cytokines. JAK inhibitors are attractive candidates for psoriasis and its use in psoriasis has recently been extensively reviewed^52^. Here, we showed that BBR decreases both total and phosphorylated JAK1/2 protein levels in keratinocytes. Moreover, BBR enhances the effects of JAK1/2 inhibitor on inhibition of STAT3 phosphorylation and apoptosis, suggesting that BBR inhibits STAT3 activation through downregulating JAK (Fig. 7j). In line with this, treatment with BBR also counteracts the effect of IL-6 on STAT3 activation, which is also mediated by JAK. To our knowledge, this is the first report that BBR could inhibit STAT3 activation by repressing expression of JAK1/2. It was reported that BBR could downregulate the phosphorylation levels of JAKs in colorectal cancer and CD4^+^ T cells^44,49^. In contrast, no change of total JAK proteins was observed in these cells, suggesting that the mechanisms by which BBR inhibits JAKs are different in different cell types.

We found that neither protein degradation nor transcription of JAK1/2 was affected by BBR treatment, suggesting that BBR may inhibit the translation of JAK1/2. MicroRNAs (miRNAs) are evolutionary conserved, small noncoding RNA molecules that are involved in post-transcriptional repression of targets^53^. Multiple miRNAs are dysregulated in psoriasis and some of them have been shown to regulate keratinocyte differentiation, proliferation, and T-cell-mediated immune dysfunction^54^. Emerging evidences suggest that BBR exerts its pharmacological effects such as anticancer and anti-inflammatory via regulation of miRNAs^55^. Therefore, it is possible that the inhibitory effect of BBR on JAK1/2 expression is through modulating miRNAs, but further studies need to be conducted to test this hypothesis.

In summary, we demonstrated that CDC6 is upregulated in epidermis in lesional skin of psoriasis. CDC6 could be induced by STAT3 signaling and is required for cell proliferation in keratinocytes. Moreover, BBR could decrease CDC6 expression and inhibit proliferation of keratinocytes via suppressing the JAK-STAT3 pathway. We also revealed that BBR could inhibit imiquimod-induced STAT3 action, CDC6 upregulation and psoriasis-like skin lesions in mice. These results suggest that BBR and its derivatives may serve as a promising therapeutic agent for psoriasis. Further clinical trials are needed to confirm its effectiveness in the therapy of psoriasis.

## Supplementary information

Figure S1

Figure S2

Figure S3

Figure S4

Figure S5

Figure S6

Supplementary Table 1

Supplementary Table 2

Supplementary Table 3

Supplementary figure legends
