# Supplementary figures and images for: Berberine downregulates CDC6 and inhibits proliferation via targeting JAK-STAT3 signaling in keratinocytes
Source: Cell Death Dis. 2019 Mar 20;10(4):274. doi: 10.1038/s41419-019-1510-8 (PMC6426889; doi:10.1038/s41419-019-1510-8)

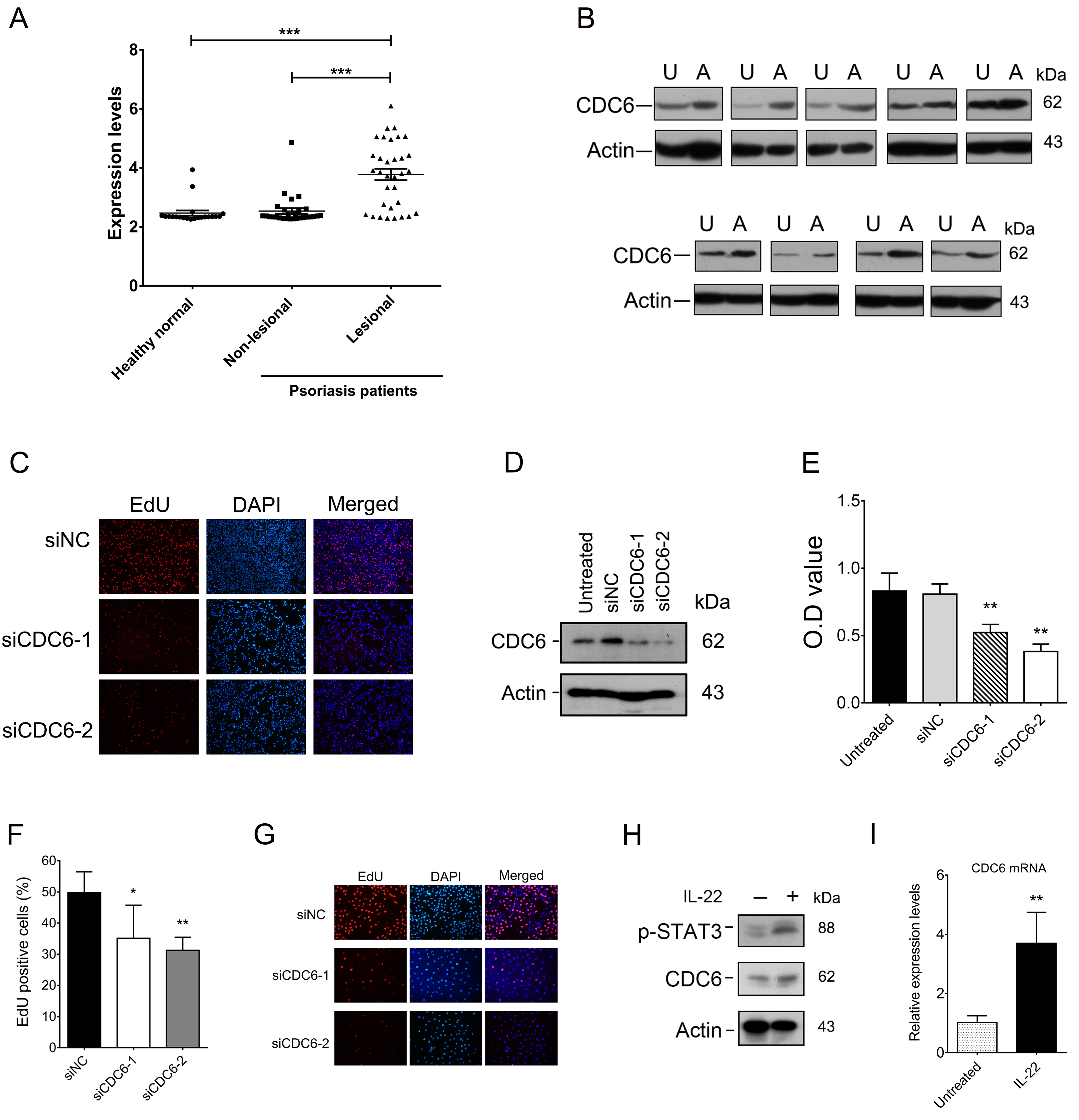

Supplement: Supplementary file 1 — Figure S1 [file 41419_2019_1510_MOESM1_ESM.jpg]

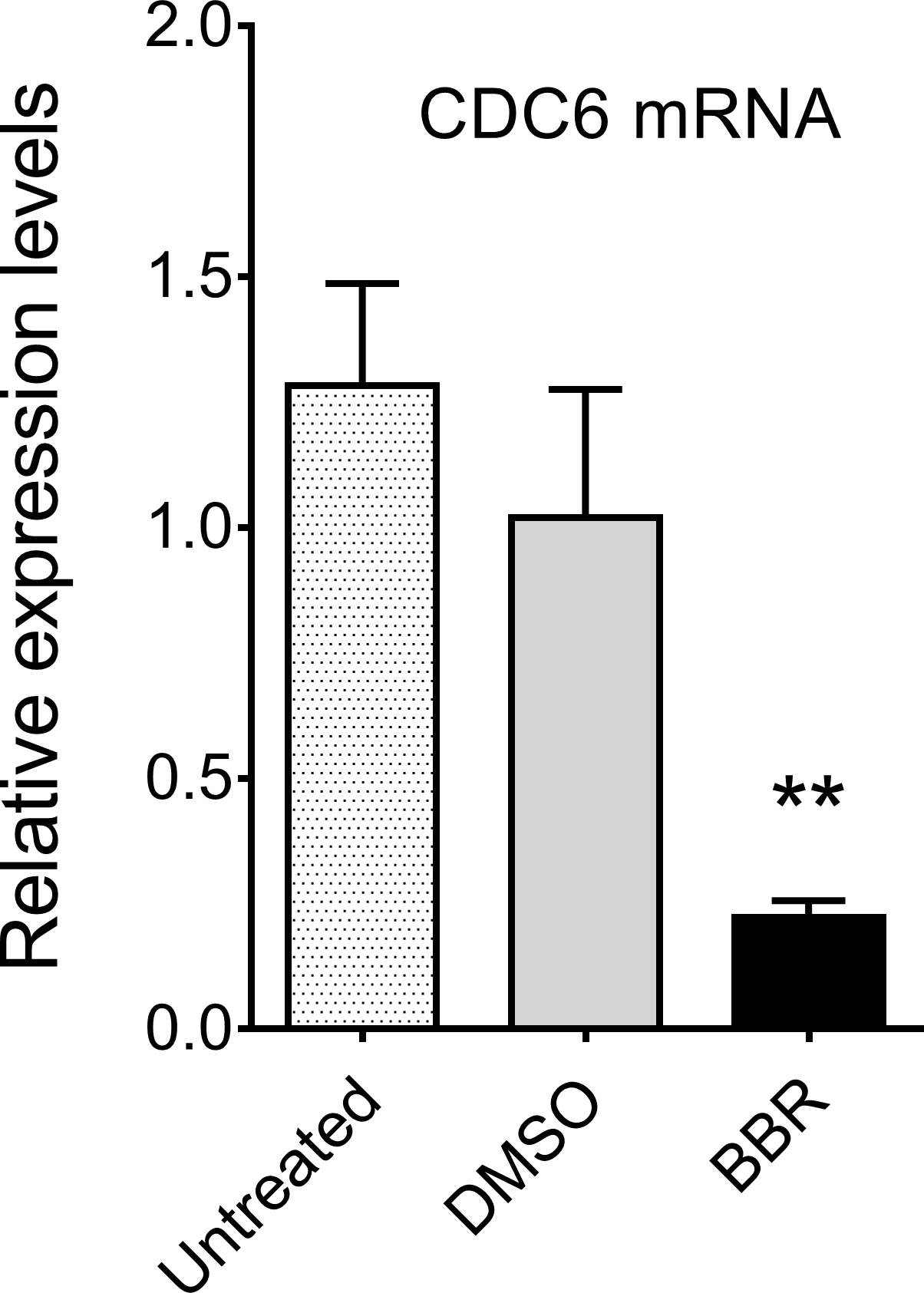

Supplement: Supplementary file 2 — Figure S2 [file 41419_2019_1510_MOESM2_ESM.jpg]

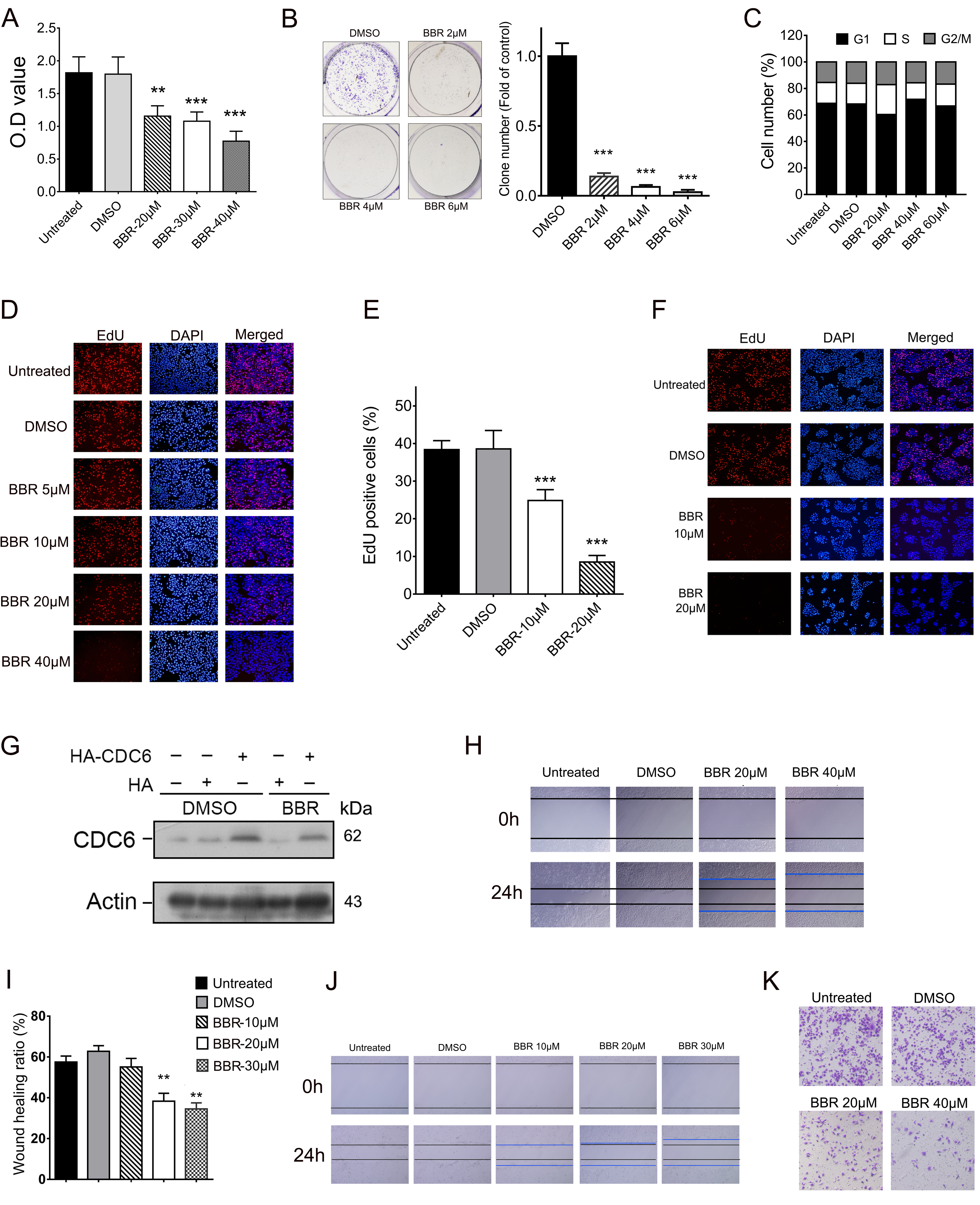

Supplement: Supplementary file 3 — Figure S3 [file 41419_2019_1510_MOESM3_ESM.jpg]

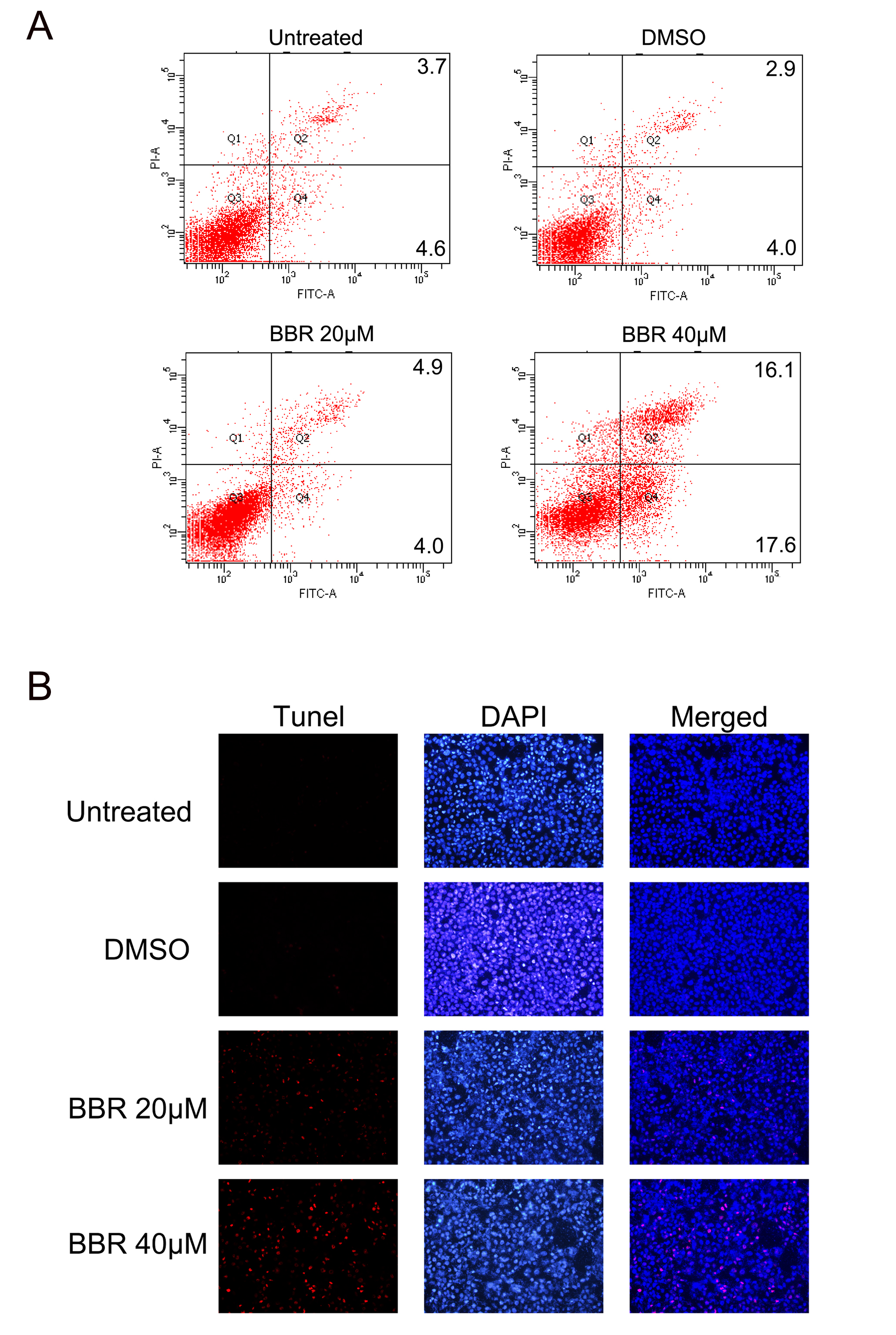

Supplement: Supplementary file 4 — Figure S4 [file 41419_2019_1510_MOESM4_ESM.jpg]

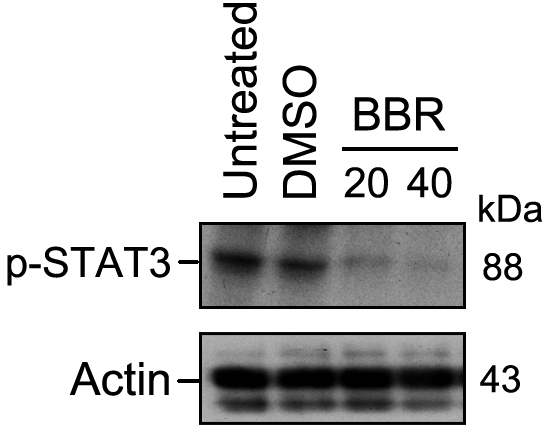

Supplement: Supplementary file 5 — Figure S5 [file 41419_2019_1510_MOESM5_ESM.jpg]

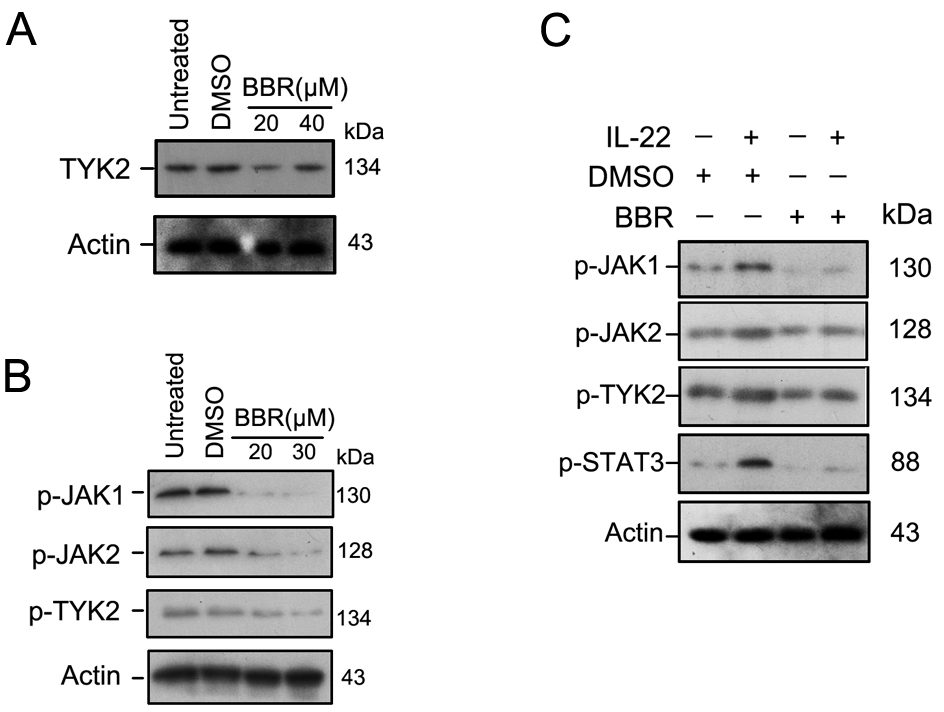

Supplement: Supplementary file 6 — Figure S6 [file 41419_2019_1510_MOESM6_ESM.jpg]
