# Supplementary material for: Berberine downregulates CDC6 and inhibits proliferation via targeting JAK-STAT3 signaling in keratinocytes
Source: Cell Death Dis. 2019 Mar 20;10(4):274. doi: 10.1038/s41419-019-1510-8 (PMC6426889; doi:10.1038/s41419-019-1510-8)
Supplement: Supplementary file 7 — Supplementary Table 1 [file 41419_2019_1510_MOESM7_ESM.pdf]

Supplementary Table 1. Sequence of siRNAs

**Name**	**Sequence （5’ - 3’）**
siNC	UUCUUCGAACGUGUCACGUTT
siCDC6#1	GACAATCAGCTGACAATTA
siCDC6#2	AGGCACTTGCTACCAGCAA
