# Supplementary material for: Berberine downregulates CDC6 and inhibits proliferation via targeting JAK-STAT3 signaling in keratinocytes
Source: Cell Death Dis. 2019 Mar 20;10(4):274. doi: 10.1038/s41419-019-1510-8 (PMC6426889; doi:10.1038/s41419-019-1510-8)
Supplement: Supplementary file 8 — Supplementary Table 2 [file 41419_2019_1510_MOESM8_ESM.pdf]

Supplementary Table 3. Primers used for real-time PCR

Name	Forward primer (5'→3')	Reverse primer (5'→3')
KRT1	AGAGTGGACCAACTGAAGAGT	ATTCTCTGCATTTGTCCGCTT
KRT10	ATGTCTGTTCGATACAGCTCAAG	CTCCACCAAGGGAGCCTTTG
CCL20	TGCTGTACCAAGAGTTTGCTC	CGCACACAGACAACTTTTTCTTT
IL-8	TTTTGCCAAGGAGTGCTAAAGA	AACCCTCTGCACCCAGTTTTC
CXCL5	AGCTGCGTTGCGTTTGTTTAC	TGGCGAACACTTGCAGATTAC
CXCL1	GCCAGTGCTTGCAGACCCT	GGCTATGACTTCGGTTTGGG
CXCL16	CCCGCCATCGGTTCAGTTC	CCCCGAGTAAGCATGTCCAC
IL-18	TCTTCATTGACCAAGGAAATCGG	TCCGGGGTGCATTATCTCTAC
IVL	TCCTCCAGTCAATACCCATCAG	CAGCAGTCATGTGCTTTTCCT
KRT5	TGACCTCCGCAACACCAAG	CAGATTGGCGCACTGTTTCTT
KRT14	TGAGCCGCATTCTGAACGAG	GATGACTGCGATCCAGAGGA
S100A7	ACGTGATGACAAGATTGACAAGC	GCGAGGTAATTTGTGCCCTTT
S100A8	ATGCCGTCTACAGGGATGAC	ACTGAGGACACTCGGTCTCTA
S100A9	GGTCATAGAACACATCATGGAGG	GGCCTGGCTTATGGTGGTG
SKALP	CACGGGAGTTCCTGTTAAAGG	TCTTTCAAGCAGCGGTTAGGG
CDC6	GCCGAACTAGAACAGCATCTT	GCCGAACTAGAACAGCATCTT
CyclinD1	CTCTGTGGAAGGCAGTTCAAA	TCAGGTAATCCCACCTTGTGTT
JAK1	CCACTACCGGATGAGGTTCTA	GGGTCTCGAATAGGAGCCAGGAGA
JAK2	TCTGGGGAGTATGTTGCAGAATCA	CATGGTTGGGTGGATACCGACATCC
GAPDH	ACAACAGCCTCAAGATCATCAG	GGTCCACCACTGACACGTTG
