# Supplementary material for: Berberine downregulates CDC6 and inhibits proliferation via targeting JAK-STAT3 signaling in keratinocytes
Source: Cell Death Dis. 2019 Mar 20;10(4):274. doi: 10.1038/s41419-019-1510-8 (PMC6426889; doi:10.1038/s41419-019-1510-8)
Supplement: Supplementary file 9 — Supplementary Table 3 [file 41419_2019_1510_MOESM9_ESM.pdf]

Supplementary Table 3. Antibodies used in Western Blot

**Name**	**Corportation**	**Cat Number**
rabbit anti- Cytochrome C	Cell Signaling Technology	11940
mouse anti- P- STAT3（Try705）	Cell Signaling Technology	9138
rabbit anti- P-AKT（Thr308）	Cell Signaling Technology	4056
rabbit anti- P- (Erk1/2) (Thr202/Tyr204)	Cell Signaling Technology	9101
mouse anti-STAT3	Abcam	Ab5073
rabbit anti-cleaved caspase-3	Cell Signaling Technology	9661
rabbit anti- PARP	Cell Signaling Technology	9532
rabbit anti-CDC6	Cell Signaling Technology	3387
rabbit anti-MCM2	Cell Signaling Technology	3619
rabbit anti-MCM4	Cell Signaling Technology	12973
mouse anti-β-actin	Santa Cruz Biotechnology	sc-58673
mouse anti-ORC2	Abcam	ab31930
rabbit anti-PCNA	Abcam	ab18197
rabbit anti-H3	Abcam	ab1791
rabbit anti-CDK4	Abcam	ab108357
rabbit anti-CDK6	Abcam	ab124821
rabbit anti-p-RB	Abcam	ab47763
rabbit anti-RB	Abcam	ab181616
rabbit anti-Cyclin D1	Abcam	ab134175
mouse anti-JAK1	Santa Cruz Biotechnology	sc-376996
mouse anti-JAK2	Santa Cruz Biotechnology	sc-390539
rabbit anti-TYK2	Cell Signaling Technology	14193
rabbit anti-P-TYK2 (Tyr1054/1055)	Cell Signaling Technology	68790
rabbit anti-P-JAK1 (Tyr1034/1035)	Cell Signaling Technology	74125
rabbit anti-P-JAK2 (Tyr1007/1008)	Cell Signaling Technology	3771
