# Supplementary material for: Berberine downregulates CDC6 and inhibits proliferation via targeting JAK-STAT3 signaling in keratinocytes
Source: Cell Death Dis. 2019 Mar 20;10(4):274. doi: 10.1038/s41419-019-1510-8 (PMC6426889; doi:10.1038/s41419-019-1510-8)
Supplement: Supplementary file 10 — Supplementary figure legends [file 41419_2019_1510_MOESM10_ESM.docx]

**Supplementary figure legends**

Figure S1. CDC6 is upregulated in skin lesions of psoriasis and is required for proliferation of keratinocytes. A. Expression analysis of CDC6 in clinical psoriasis samples using public GEO data (GEO ascension GDS3539). ***: p < 0.001. B. Western blot analysis of CDC6 in adjacent unaffected nonlesional (U) and affected lesional (A) skin from patients of psoriasis. C. Representative images of EdU assays on HaCaT cells transfected with indicated siRNAs. D. HEKn cells were transfected with 50 nM negative control (siNC) or CDC6 targeting siRNAs (siCDC6). 72 h later, the CDC6 protein levels were analyzed by Western blot. E-G. HEKn cells were transfected with indicated siRNAs for 72 h and cell proliferation, DNA replication was determined by CCK8 assays (E), EdU assays (F and G). *: p < 0.05, **: p < 0.01, compared to that of NC cells. H-I. HEKn cells were treated with 100ng/ml IL-22 for 48 h and protein levels of CDC6 were analyzed by Western blot (H) and mRNA was analyzed by qRT-PCR (I). **: p < 0.01,

Figure S2. BBR downregulates CDC6 mRNA levels in HEKn cells. HEKn cells were treated with or without 20 μM BBR for 48 h and qRT-PCR was employed to detected CDC6 expression. **: p < 0.01, compared to that of DMSO treated cells.

Figure S3. BBR inhibits proliferation and migration of keratinocytes. A. CCK8 assays of HEKn cells treated with or without BBR for 72 h. B. Colony formation assays of HEKn cells treated with or without BBR. C. HEKn cells were treated with or without BBR for 48 h and cell cycle distribution were analyzed by flow cytometry. D. Representative images of EdU assays on HaCaT cells treated with or without BBR for 24 h. E-F. EdU assays on HEKn cells treated with or without BBR for 24 h. G. HaCaT cells were transfected with HA tagged CDC6 expression plasmid or control plasmid (HA). 48h later, cells were treated with or without 40 μM BBR for another 24 h and Western blot was employed to detected CDC6 expression. H. Representative images of wound healing assays on HaCaT cells treated with or without BBR. I-J. Wound healing assays on HEKn cells treated with or without BBR. K. Representative images of transwell assays on HaCaT cells treated with or without BBR. **: p < 0.01, ***: p < 0.001, compared to that of DMSO treated cells.

Figure S4. A. HEKn cells were treated with or without BBR for 72 h and cell apoptosis was assessed by flow cytometry. B. Representative images of TUNEL assays on HaCaT cells treated with or without BBR for 72 h.

Figure S5. HEKn cells were treated with different concentration of BBR for 48 h and p-STAT3 levels were analyzed by Western blot.

Figure S6. A. HaCaT cells were treated with or without BBR for 48 h and total TYK2 protein levels were analyzed by Western blot. B. HEKn cells were treated with or without BBR for 48 h and indicated protein levels were determined by Western blot. C. HEKn cells were treated with or without 20μM BBR for 24 h followed by treatment with 100ng/ml IL-22 for another 24 h and indicated protein levels were determined by Western blot.
